# The Leukemoid Reaction in Severe Alcoholic Hepatitis: A Case Report

**DOI:** 10.7759/cureus.54039

**Published:** 2024-02-11

**Authors:** Siva Reddy, Sachin Agrawal, Sunil Kumar, Sourya Acharya

**Affiliations:** 1 Medicine, Jawaharlal Nehru Medical College, Datta Meghe Institute of Higher Education and Research, Wardha, IND

**Keywords:** illness, clinicopathologic, leukocytosis, leukemoid reaction, alcoholic hepatitis

## Abstract

Alcoholic hepatitis (AH) is a clinicopathologic illness caused by excessive alcohol abuse and is a precursor of cirrhosis. The leukemoid reaction (LR) is characterized by a strikingly raised granulocyte count of 40,000-50,000 cells/mm^3^. The LR usually suggests an acute inflammatory reaction. It is usually mistaken for chronic myeloid leukemia. The initial phase of leukocytosis occurs due to the releasing of cells from the bone marrow with more immature cells, causing a left upper shift in the ratio of immature to mature neutrophils and macrophages. The LR is usually seen in cases of leukemia but is rare to present in alcohol hepatitis. Excessive alcohol use causes AH in persons with or without underlying chronic liver disease. In severe AH, leukemoid responses have been associated with very poor prognosis and short-term mortality. We describe a case of a 35-year-old male with severe AH with an LR.

## Introduction

Alcoholic hepatitis (AH) is a clinicopathological illness that presents as acute to subacute liver failure, clinical icterus, coagulopathy, liver enzyme elevation pattern, hepatic encephalopathy, variceal bleeding, and sepsis. Finally, multiorgan failure may ensue [1]. A leukemoid response is a severe form of leukocytosis caused by a mechanism outside the bone marrow. The difference between leukocytosis and leukemoid reaction (LR) is typically set at total leucocyte counts between 40-50,000/mm^3^. Infections, stress, and cancer are also common causes of LRs [2]. When the leukocytosis is larger than 30,000/mm^3^ or if anemia or thrombocytopenia are present, malignancy must be ruled out. Although a leukocytosis of 15-18,000/mm^3^ is common in AH, LRs are uncommon in the setting of AH, with only a few data available in scientific literature. AH-related LRs indicate a bad prognosis and a negative outcome [3].

## Case presentation

**Figure 1:**
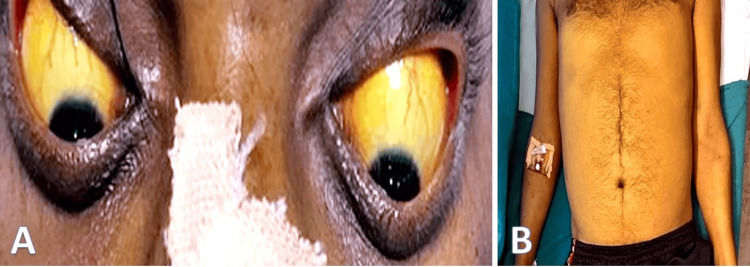
(A) Yellowish discoloration of sclera; (B) Yellowish discoloration over whole body

A 35-year-old male was brought to the emergency department by his relatives with a complaint of two episodes of blood in stools, associated with abdominal pain and loss of appetite for three days. He was a chronic alcoholic for 10 years, two drinks per day, and his last intake was one month back. The patient was conscious on examination and oriented to time, place, and person. His BP was 120/70 mmHg, pulse was 88 beats per minute, jugular venous pressure was normal, and respiratory rate was 16 cycles per minute. Icterus was present, and cyanosis, clubbing, and edema were absent. Per abdomen, examination revealed tender hepatomegaly 2 cm below the right costal margin (Figures 1A-1B).

Investigations were done for blood tests, including complete blood count, peripheral smear, and liver function test, and the results are mentioned in Table 1.

**Table 1:** Laboratory blood test results

Sr. No	Name of Investigation	Patient Value	Reference Value
1	Haemoglobin	9	13.1-17.5 gm/dL (for male)
2	Total leukocyte count (TLC)	68,000	4000-11000 cells/cumm
3	Differential leucocyte count (DLC)	00	00
4	Neutrophils	50	40-60%
5	band (young neutrophils) forms	20	0-3%
6	Lymphocytes	18	20-40%
7	Monocytes	02	02-08%
8	Eosinophils	02	01-04%
9	Basophils	00	0.5-1%
10	Myeloblasts	0	0
11	Promyelocytes	01	0
12	Metamyelocytes	05	0
13	Serum bilirubin (Total)	18.2	1.2 mg/dl
14	Bilirubin direct	12.4	<0.3 mg/dl
15	Bilirubin indirect	5.8	0.2 to 1.2 mg/dL
16	C-reactive protein	22	<1.0 mg/dl
17	Aspartate transferase (AST)	390	10-44.75 U/L
18	Alanine transaminase (ALT)	152	13.62-71.40 U/L

Peripheral smear finding was suggestive of an LR. No hemiparasites were seen. Prothrombin time was 19 (reference value - 11 to 13.5 seconds), prothrombin time was 2.8 (reference value - 2.0 to 3.0), with an activated partial thromboplastin time of 40 s. Maddrey's discriminatory function score was 48 with a Child-Pugh-Turcotte of 7, and the Model for End-Stage Liver Disease (MELD) was 20. Blood culture and urine culture did not reveal any growth. Chest radiography (CXR) was normal. The leukocyte alkaline phosphatase (LAP) score was 380. Janus kinase 2 (JAK-2) mutation analysis was negative. The patient was started on prednisolone 1 mg/kg body weight, and there was a decrease in bilirubin and total leukocyte count (TLC) after one week and tab. Prednisolone was continued for another three weeks and was tapered over the next three weeks. The patient also received an injection of Vitamin K, 5 units of fresh frozen plasma, intravenous thiamine, antacids, and ursodeoxycholic acid. The patient improved, bilirubin came down to 4 mg/dl. TLC at the time of discharge was 16,000/mm^3^. After one-month follow-up, TLC was 8500/mm^3^.

**Figure 2:**
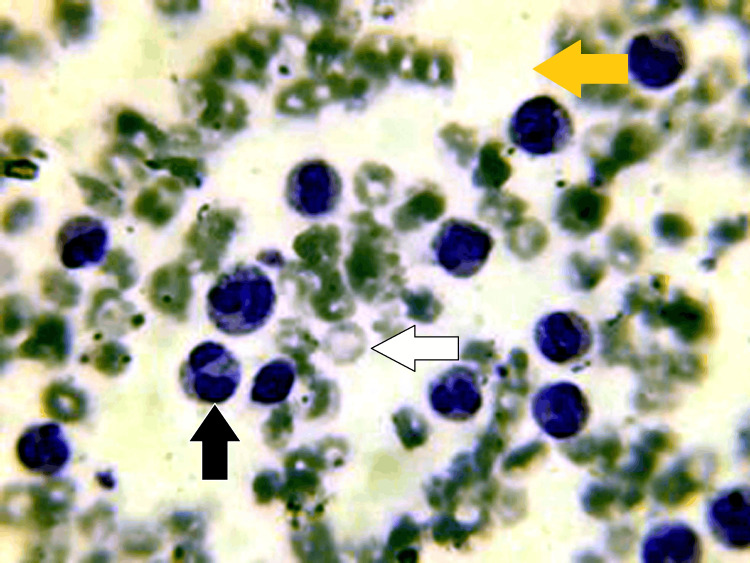
Peripheral smear findings: Peripheral smear stained with Leishman stain (oil immersion view: 100x) Peripheral smear also has neutrophilic leucocytosis, shifting to band forms (yellow arrow) and metamyelocytes (white arrow). Neutrophils also show toxic granule (black arrow).

Figure 2 shows normocytic normochromic RBCs. The TLC is raised. Mild thrombocytopenia is present (Figure 2). Opinion from the consultant given peripheral smear findings are suggestive of a LR.

## Discussion

AH is a severe manifestation of alcohol-related liver disease, often associated with a high mortality rate. While leukocytosis is a common finding in AH, the occurrence of an LR is rare but indicative of a poor prognosis. Our case report adds to the limited literature on this topic, highlighting the importance of recognizing and managing this severe complication. Several previous case reports have documented the association between AH and LR, albeit in a relatively small number of cases [2-5]. For instance, Morales et al. reported a case of AH with LR following surgery, emphasizing the potential exacerbation of liver pathology in the perioperative period [2]. Similarly, Larvol et al. and Petracca et al. described cases of AH complicated by LR, further underlining the grave prognosis associated with this presentation [3,4].

In line with these reports, our case illustrates the clinical and laboratory features of AH with LR, including markedly elevated leukocyte counts, characteristic peripheral smear findings, and severe liver dysfunction. Importantly, the prompt initiation of corticosteroid therapy, guided by established prognostic indicators such as the discriminant function score, was associated with a favorable outcome in our patient [6-10]. The pathophysiology of LR in AH remains incompletely understood but likely involves the dysregulated production of proinflammatory cytokines such as granulocyte-macrophage colony-stimulating factor (GM-CSF), tumor necrosis factor-alpha (TNF-α), and interleukins [6]. These cytokines, released in response to liver injury and inflammation, promote the proliferation and differentiation of granulocyte precursors in the bone marrow. This leads to the characteristic left shift in neutrophil maturation seen in LR.

While corticosteroids have been shown to improve survival in AH, their efficacy in cases with LR warrants further investigation. Singal et al. demonstrated the benefits of corticosteroid therapy in AH, with improved short-term survival and reduced risk of hepatorenal syndrome [11]. However, the optimal timing and duration of corticosteroid treatment in AH with LR remain unclear and require prospective studies for validation.

## Conclusions

In conclusion, the case of severe AH is rarely associated with the LR. It increases short-term mortality. Before confirming the diagnosis, myeloproliferative disorders should be ruled out by appropriate investigations. The LR has been associated with poor outcomes in severe AH; our patient improved with steroids. Further studies should be done to evidently prove whether LR should be used as a criterion for starting steroids. Before starting corticosteroids, sepsis, malignancies, and other infections should be ruled out.
